# Case Report: Spontaneous perforation of a bicornuate uterus with concomitant sarcoma

**DOI:** 10.12688/f1000research.25961.2

**Published:** 2020-11-13

**Authors:** Soobin Yim, Inji Yeo, Myunghwa Lee, Kyu-Sang Kyeong, Hye-yon Cho, Jung Bae Kang, Min Sun Kyung

**Affiliations:** 1 Department of Obstetrics & Gynecology, Hallym University Dongtan Sacred Heart Hospital, Hwaseong, South Korea

**Keywords:** Bicornuate uterus, Uterine rupture, Uterine sarcoma

## Abstract

A 47-year-old nulliparous, virginal woman presented to the emergency department with acute abdominal pain. Emergency pelvic ultrasound and abdominal CT were taken, which showed a significant amount of hemoperitoneum and a bicornuate uterus with about 18cm x 10cm mass on left uterus. Since the mass had increased vascularity and irregular margins, we thought that the mass could be a uterine sarcoma. MRI and PET/CT were taken additionally for oncologic evaluation before surgery. Intra operative findings showed a ruptured bicornuate uterus with a large mass within the left uterine horn. Total

abdominal hysterectomy with bilateral salpingo-oophorectomy was performed. Pathologic analysis confirmed an undifferentiated uterine sarcoma. She was treated with 6 cycles of chemotherapy(etoposide, ifosfamide, cisplatin) postoperatively. Chest and abdomen CT for follow up after chemotherapy showed no sign of cancer recurrence. We suggest a bicornuate uterus with concomitant sarcoma should be concerned as a possible cause of uterine rupture by reviewing this case.

## Introduction

Spontaneous uterine rupture occurs most commonly with labor and delivery ^1^. When it does occur, the most common cause of rupture is dehiscence of a previous transmyometrial surgical incision, such as that from a cesarean section scar ^2^. Spontaneous rupture of a uterus without a previous surgical scar is very uncommon and significantly less is known ^2^.

Bicornuate uterus is a common type of congenital uterine malformation: it takes the form of a double uterus with a single cervix and vagina ^3^. Implantation of the zygote in a rudimentary horn of bicornuate uterus is considered an independent risk factor for uterine rupture. Because when a zygote is implanted in a horn of a bicornuate uterus, it is unable to expand as a normal uterus does to accommodate a growing fetus ^4^. The walls of the anomalous uterus tend to become abnormally thin as pregnancies advance, and the uterine rupture can happen ^5^.

Uterine sarcoma is a rare and aggressive soft tissue neoplasm in women of all ages. It usually presents with abdominal or pelvic pain, vaginal bleeding; sarcoma does not typically cause uterine rupture and hemoperitoneum. To our knowledge, there have been only five cases reported in the literature that describe a uterine sarcoma presenting with rupture and induced hemoperitoneum ^6^.

Herein, we report a case from diagnosis to surgical treatment of a 47-year-old woman with bicornuate uterus who had no previous history of spontaneous uterine rupture or uterine surgery. We hypothesize that as the uterine sarcoma advance, uterine rupture can occur as a result of congenitally malformed, bicornuate uterus. Through this case, we suggest that a bicornuate uterus with concomitant sarcoma should be concerned as a possible cause of uterine rupture when a woman presents with hemoperitoneum in the setting of a pelvic mass and uterine anomaly with intact ovaries detected on imaging.

## Case report

A 47-year-old nulliparous, virginal woman presented to the emergency department with a 2-day fever and acute abdominal pain. She also complained of a 2-month history of foul-smelling vaginal bleeding. She had pyrexia, with a temperature of 38°C (100.4°F), and marked tenderness of the whole abdomen. She has a past medical history significant for breast cancer, which was treated with six cycles of CAF (cyclophosphamide, [doxorubicin] Adriamycin, fluorouracil), adjuvant radiotherapy, and tamoxifen for 4 years; the doses are uncertain since she had taken those therapies in the other institutes. She has not had a gynecological evaluation for over 8 years since the end of her breast cancer treatment, and she had discontinued her tamoxifen.

Laboratory investigation demonstrated a hemoglobin level of 12.3 g/dL (normal range, 12–16 g/dL), a white blood cell count of 12,600/mm ^3 ^(normal range, 4,000–10,000/mm ^3^) and a C-reactive protein of 157.9 mg/L (normal range, 0.1–5.0 mg/L). Mild elevation of her liver enzymes was observed, with a total bilirubin of 2.2 mg/dL (normal range, 0.2–1.4 mg/dL) and a direct bilirubin of 0.7 mg/dL (normal range, 0.0–0.5 mg/dL).

A dynamic CT scan of the liver was performed to evaluate for biliary infection as this patient had been known to have a history of gallbladder stones for years along with complaints of upper abdominal pain. The CT scan revealed a 16-cm uterine mass and increased free fluid in the pelvic cavity, including both paracolic gutters, the perihepatic space, and the perisplenic space. Consequently, she was referred to our obstetrics and gynecology department.

**Figure 1.**
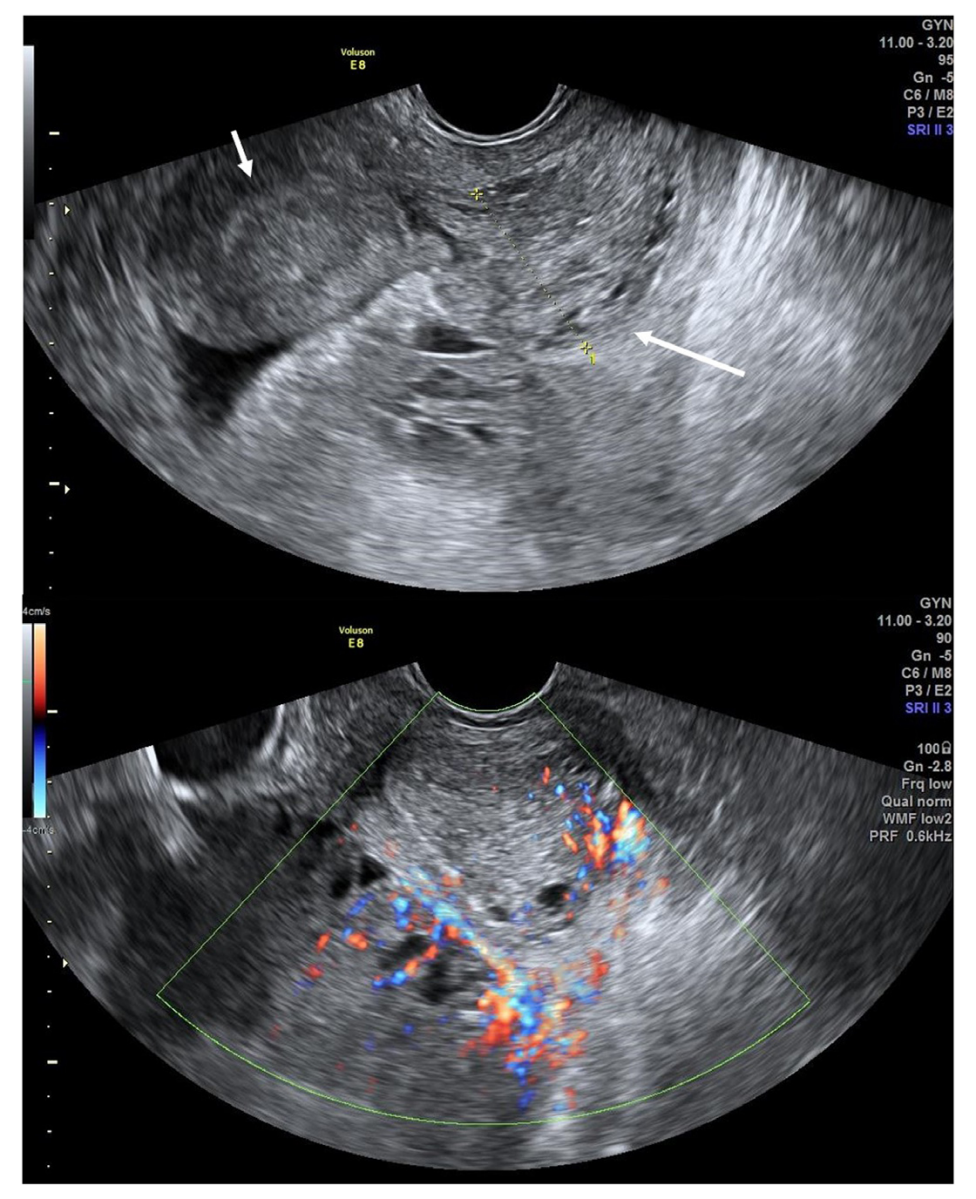
Transrectal ultrasonography. Ultrasonography shows a 1.5-cm-thick endometrial layer in the right uterus (short arrow) and up to 2.69 cm in the left uterus (long arrow). The mass was connected to the left uterus and appeared to be extending into the vaginal cavity. Internal blood flow was increased.

Transrectal ultrasonography showed a bicornuate uterus and an 18.6- × 9.1-cm mass with heterogeneous echogenicity within the left uterus. The mass appeared to extend through the cervix into the vaginal cavity. Its irregular-shaped margins and increased blood flow suggested the possibility of malignancy ( Figure 1). Because her vital signs were stable (aside from fever), the patient received an oncological evaluation and antibiotic treatment before surgery. Since F-18 FDG were ordered and needed time to arrive, PET/CT were planned two days later, and the surgery was planned for the next day. Antibiotics (piperacillin 4g, tazobactam 0.5g, metronidazole 0.5g) were injected intravenous every 8 hours, and administered for about 48 hours until just before surgery.

**Figure 2.**
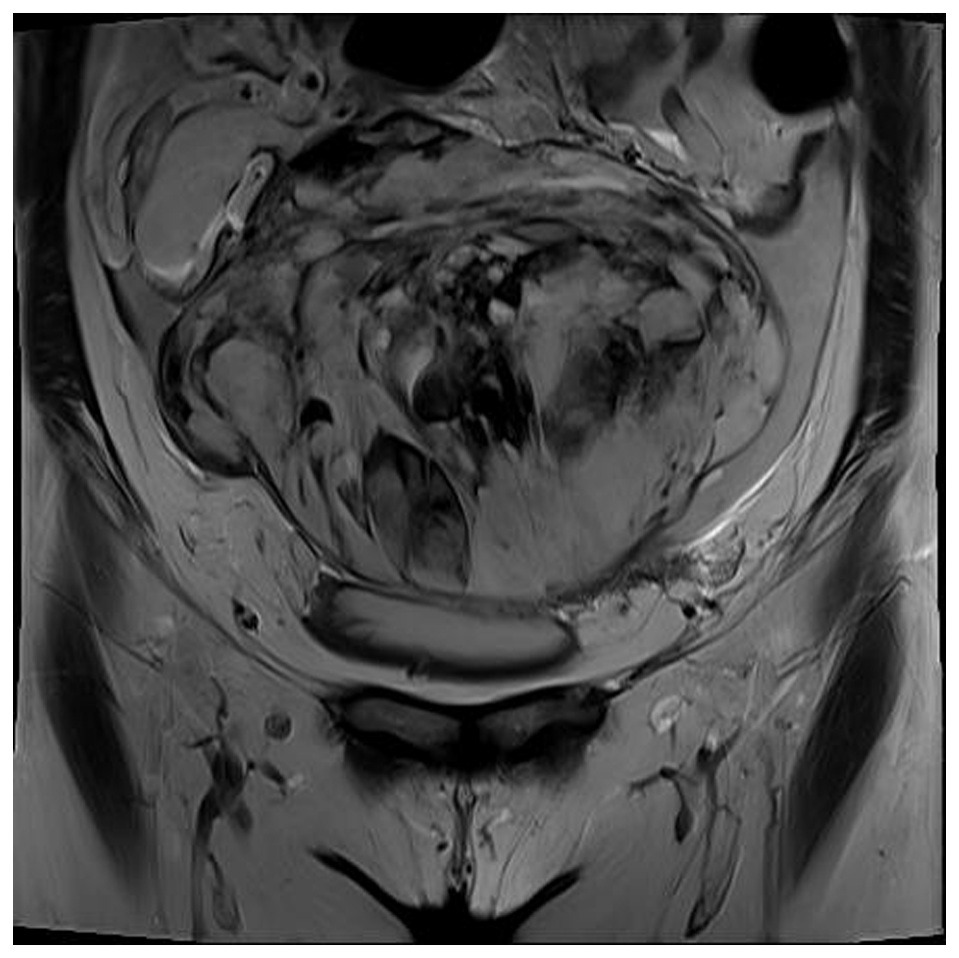
Magnetic resonance imaging (coronal view, T2 W1). This scan shows an approximately 15- × 9- × 17-cm mass with mixed high and low signal intensity and irregular margins in the lower abdomen on coronal view.

Pelvic MRI was performed on the day after the patient’s initial presentation, revealed underlying uterus didelphys with an approximately 15- × 9- × 17-cm mass with mixed signal intensity in the lower abdominal area ( Figure 2) and an approximately 6- × 2.7- × 3-cm mass of the left cervix and lower uterine body on T2-weighted imaging. These MRI findings suggested the possibility of hemoperitoneum or cancer peritonei due to rupture of (1) endometrial cancer, (2) uterine sarcoma, or (3) large myoma with degeneration.

**Figure 3.**
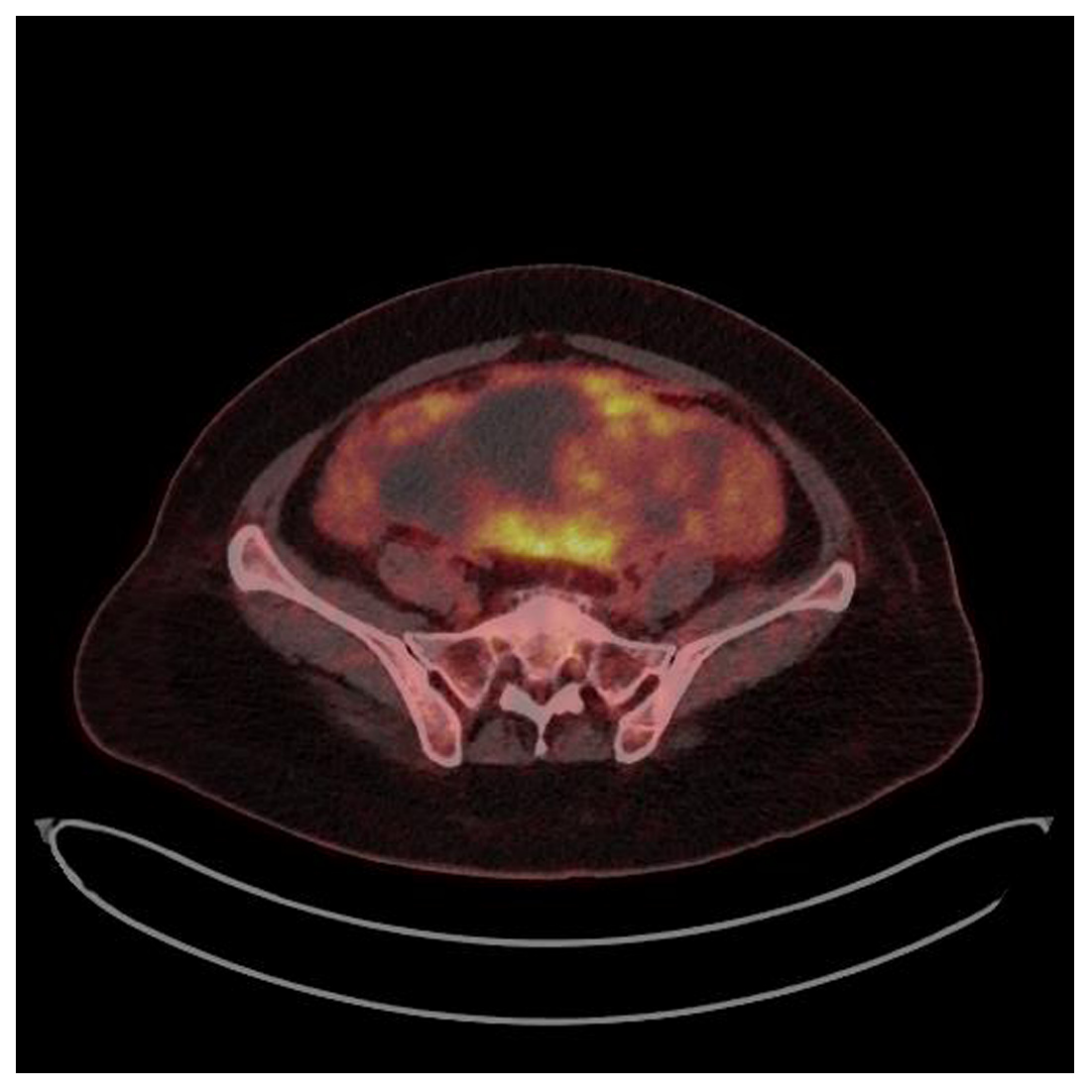
Positron emission tomography/computed tomography. An approximately 13-cm intensely hypermetabolic hemorrhagic mass is noted in the pelvic cavity, suggestive of a subserosal uterine mass possibly connected with a cervical lesion. There are regions of hypermetabolic peritoneal thickening and nodules with a small amount ascites.

A whole-body PET/CT scan was ultimately performed (two days after presentation) and showed an intensely hypermetabolic subserosal mass with accompanying hemorrhage and possible rupture; further, hypermetabolic peritoneal nodules and peritoneal infiltration with ascites were observed ( Figure 3).

**Figure 4.**
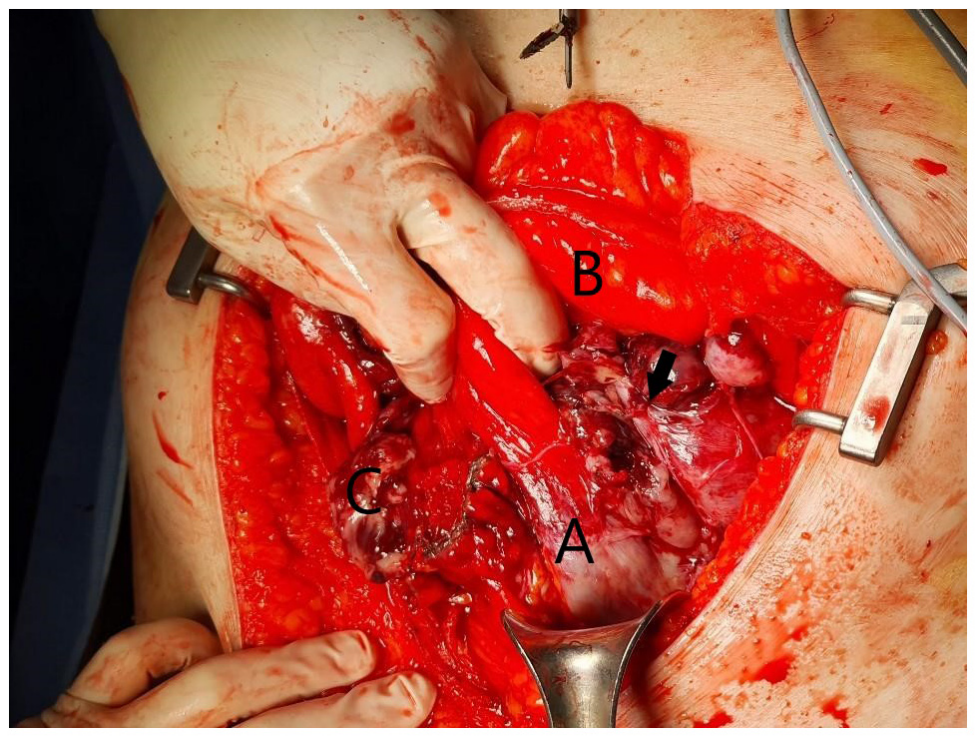
The arrow is pointing the ruptured portion of left uterine horn of the bicornuate uterus. Rupture length was about 3cm. Mesentery of small bowel was partially adhered to the uterine mass. A significant amount of hemoperitoneum was observed. Estimated blood collection in cul-de-sac was 1000 to 1500 mL. (A) Left uterus (B) Small bowel (C) Mass.

The patient underwent diagnostic laparotomy three days after presentation. Surgical exploration revealed a ruptured bicornuate uterus with a large mass within the left uterine horn ( Figure 4). The mesentery of the small bowel and appendix were partially adhered to the uterine mass. Total abdominal hysterectomy with bilateral salpingo-oophorectomy was performed, and invasive cancer implants within the bowel were removed. Estimated surgical blood loss was approximately 1500 mL.

Pathological analysis confirmed an undifferentiated uterine sarcoma with tumor size up to 23 × 13 × 6 cm. Because a tumor in her left fallopian tube and one of her bowel mass implants tested positive for cancer, she was diagnosed with FIGO Stage IIIA (from the International Federation of Gynecology and Obstetrics cancer staging system) ^7^.

Postoperatively, the patient was treated with a combination of etoposide (150 mg/m ^2^ for 3 days), ifosfamide (1.5 g/m ^2^ for 3 days), and cisplatin (70mg/m ^2^ for 1 day) once every 3 weeks. Chest and abdomen CT were performed for follow up 2 weeks after. There were no sign of cancer recurrence in the chest and abdominal CT.

## Discussion

The uterus is pear shaped and consists of two major but unequal parts. The upper, larger portion is the body or corpus, whereas the lower smaller cervix projects into vagina. The bulk of the uterine body is muscle. Almost the entire posterior wall of the uterus is covered by serosa, that is, visceral peritoneum. Uterine rupture can present with both complete rupture involves the full thickness of the uterine wall and incomplete rupture occurs with the visceral peritoneum remains intact. Both types of uterine rupture are rare but serious events. Several risk factors have been identified. The most common risk factor is previous transmyometrial surgical incision, typically the result of a cesarean section ^1, 2^. Other significant risk factors for uterine rupture include oxytocin-induced labor, antepartum fetal death, and first trimester miscarriages ^8^. There have been several reported cases of uterine rupture in Müllerian anomalies where the zygote implants within a rudimentary horn. Higher rates of uterine rupture have been reported in patients with Müllerian duct abnormalities who elect to undergo a trial of labor after cesarean delivery when compared with patients without Müllerian duct abnormalities, suggesting that these anomalies may be an independent risk factor for uterine rupture ^5^.

The uterus is formed by the fusion of two paramesonephric ducts (Müllerian ducts) during embryogenesis. The separate ducts fuse into a single uterine body between the sixth and eighth weeks of gestation ^9^. Failure of complete fusion of the Müllerian ducts leads to various types of malformations of the female genital tract ^10^. The incidence of uterine anomalies is 0.06% to 38% in the general population ^11^. Bicornuate uterus is a common type of uterine malformation, taking the form of a double uterus with a single cervix and vagina. Each uterus has a single horn linked to an ipsilateral fallopian tube that faces its ovary ^3^. The bicornuate uterus often has unusually thick and strong round ligaments along with a thick vesicorectal fold running between them ^12^. This fibrous band in the form of a rectovesical ligament has a restrictive effect on the expansion of the pregnant horn of a bicornuate uterus, thereby weakening the medial aspect of the horn ^13^. Rupture of a bicornuate uterus during a growing pregnancy occurs due to inability of the malformed uterus to expand as a normal uterus would ^4^. The walls of the anomalous uteri tend to become abnormally thin as the pregnancy advances. Rudimentary horn rupture is likely to occur in the late first trimester or even in the second trimester ^3^.

Some cases are associated with leiomyoma of the uterus. Potential etiologies for spontaneous rupture of leiomyomas are degeneration or sarcomatous changes of uterine leiomyomas. The most likely mechanism behind spontaneous rupture of a leiomyosarcoma is tumor necrosis. Hemoperitoneum is the result of spontaneous rupture of the superficial vessels overlying leiomyomas ^14^ or the result of avulsion of a pedunculated myoma by trauma ^15, 16^. Uterine rupture with resulting hemoperitoneum has been described particularly for leiomyomas, and approximately 100 cases have been reported. Because uterine sarcoma is a rare neoplasm; therefore, uterine rupture as a result of sarcoma is even more unusual. A patient with uterine rupture typically presents in hypovolemic shock with severe abdominal pain that requires emergency care. Because a patient’s condition can deteriorate rapidly after uterine rupture, most patients will need immediate blood and fluid replacement therapy and an exploratory laparotomy to be done without a clear preoperative diagnosis before surgery. Most uterine sarcomas are diagnosed after surgery by postoperative biopsy ^17^.

We formulated two hypotheses regarding the etiology of uterine rupture in our patient: (1) the sarcoma itself had necrotic changes that led to uterine rupture and (2) the patient had an underlying bicornuate uterus, which exerted a restrictive effect on the ability of the myometrium to expand in the setting of a growing sarcoma.

Pelvic ultrasonography or CT imaging is commonly used for preoperative diagnosis of uterine sarcoma. Ultrasonography can also be the initial examination measure performed urgently in the setting of acute pelvic symptoms. However, ultrasonography remains insufficient to differentiate between a remodeled myoma and a sarcoma based on morphologic criteria alone. As a result, color Doppler ultrasonography can be used to measure resistance indexes which reveal a significant difference between leiomyomas (resistance index 0.01–0.59) and sarcomas (resistance index 0.06–0.41) ^18^

Ultrasonography is useful when performed properly, but it has a limitation for revealing a large mass. CT imaging is superior to ultrasonography especially for evaluation of a large mass. Also CT imaging has good diagnostic value, because extravasation of contrast enhancement can be used to evaluate for active bleeding. Further, a diagnosis of hemoperitoneum can be made by assessing the Hounsfield unit (HU), a measure of radiodensity. The attenuation values of unclotted extravascular blood usually measure between 30 and 45 HU, whereas clotted blood is between 45 and 70 HU because of its high protein content ^19^. However, a CT imaging is not sensitive enough to distinguish between a leiomyosarcoma and a necrotic leiomyoma ^20^.

Pelvic MRI is a useful diagnostic tool for the detection and characterization of uterine sarcoma, as well as for an assessment of disease staging. MRI images of uterine sarcomas generally have irregular margins and are isointense or hypointense on T1-weighted imaging (compared to the signal from the myometrium) with possible hemorrhagic zones. In T2-weighted imaging, uterine sarcomas have an intermediate-to-high signal. In contrast, leiomyomas have regular margins and low-to-intermediate signals on both T1-weighted and T2-weighted imaging. Although the radiological findings of these lesions can overlap, characteristic features can help narrow the differential diagnosis and guide adequate treatment selection and follow-up. In addition to morphologic features, diffusion-weighted imaging seems to be a potentially useful tool in the characterization of large uterine lesions ^21^.

In our patient case, it was uncertain whether the uterine rupture was due to endometrial cancer, uterine sarcoma, or degenerated myoma. The mass appeared to be extending through the cervix into the vaginal cavity, but vaginal examination and endometrial biopsy could not be performed because the patient is a virgin. The patient had stable vital signs other than pyrexia, and her pain was not severe. Therefore, we decided to perform PET/CT scan additionally to distinguish between leiomyoma and sarcoma to determine the scope of preoperative surgery.

The treatment of uterine sarcomas is surgical intervention. It includes a total hysterectomy with bilateral adnexectomy in the event of a tumor limited to the uterine body. The indication for adjuvant treatment remains a topic of debate ^22^. The prognosis for uterine sarcomas remains poor. The probability of survival at 5 years is estimated at 30%, all stages combined ^23^.

Though uncommon, uterine rupture in the setting of a bicornuate uterus with concomitant sarcoma should be highlighted as a possible cause of hemoperitoneum when a woman presents with severe abdominal pain, particularly in the setting of a pelvic mass and uterine anomaly with intact ovaries detected on imaging. CT imaging is useful when ultrasonography shows no definite evidence of uterine rupture. We recommend the use of MRI imaging to help differentiate between leiomyoma and sarcoma before surgical intervention takes place.

## Data availability

All data underlying the results are available as part of the article and no additional source data are required.

## Consent

We received written informed consent from the patient for the use and publication of the patient’s data.
