# Supplementary material for: Case Report: Spontaneous perforation of a bicornuate uterus with concomitant sarcoma
Source: F1000Res. 2020 Nov 13;9:1143. Originally published 2020 Sep 16. [Version 2] doi: 10.12688/f1000research.25961.2 (PMC7783531; doi:10.12688/f1000research.25961.2)
Supplement: Peer review history

# Peer review: Reviewer response for version 2

**DOI:** 10.5256/f1000research.30570.r74760

**Published:** 2021-01-04

**Authors:** Pallav Sengupta, Sulagna Dutta

**Affiliations:** 1 Department of Physiology, Faculty of Medicine, Bioscience and Nursing, MAHSA University, Kuala Lumpur, Malaysia; 2 MAHSA University, Kuala Lumpur, Malaysia

The authors have revised the article as per the given comments. I recommend publication of the article in its present form.

Are enough details provided of any physical examination and diagnostic tests, treatment given and outcomes?

Yes

Is the case presented with sufficient detail to be useful for other practitioners?

Yes

Is sufficient discussion included of the importance of the findings and their relevance to future understanding of disease processes, diagnosis or treatment?

Yes

Is the background of the case’s history and progression described in sufficient detail?

Yes

Reviewer Expertise:

Reproductive Endocrinology

We confirm that we have read this submission and believe that we have an appropriate level of expertise to confirm that it is of an acceptable scientific standard.

# Peer review: Reviewer response for version 2

**DOI:** 10.5256/f1000research.30570.r74759

**Published:** 2020-11-20

**Authors:** Jin-Sung Yuk

**Affiliations:** 1 Department of Obstetrics and Gynecology, Sanggye Paik Hospital, School of Medicine, Inje University, Seoul, South Korea

I think you've made a good correction.

Are enough details provided of any physical examination and diagnostic tests, treatment given and outcomes?

Yes

Is the case presented with sufficient detail to be useful for other practitioners?

Yes

Is sufficient discussion included of the importance of the findings and their relevance to future understanding of disease processes, diagnosis or treatment?

Partly

Is the background of the case’s history and progression described in sufficient detail?

Yes

Reviewer Expertise:

Endocrinologic Gynecologist

I confirm that I have read this submission and believe that I have an appropriate level of expertise to confirm that it is of an acceptable scientific standard.

# Peer review: Reviewer response for version 1

**DOI:** 10.5256/f1000research.28649.r72608

**Published:** 2020-10-23

**Authors:** Pallav Sengupta, Sulagna Dutta

**Affiliations:** 1 Department of Physiology, Faculty of Medicine, Bioscience and Nursing, MAHSA University, Kuala Lumpur, Malaysia; 2 MAHSA University, Kuala Lumpur, Malaysia

The case report on 'Spontaneous perforation of a bicornuate uterus with concomitant sarcoma' by Yim et al is describing an interesting finding. The authors have described the physical and laboratory investigations and operative and post-operative procedures well. The report can be accepted in F1000Research. However, the presentation of the literature in various sections can be improved further to improve the readability of the article.

1. Abstract: This section is well-presented. However, it is preferable to discuss some information on operative as well well as post-operative procedures with the lab investigations. Please also try to rewrite the last sentence which reads repetitive.
2. Introduction: Nicely written. The last paragraph should discuss the objective specifically, rather than mentioning the methods performed. 
3. Case Report: Well described.
4. Discussion: Please start with the discussion of normal uterine structures and development (as done in the second paragraph) and continue with the structural abnormalities and so on. The rest has been discussed well.
5. The authors are requested to provide the ethical approval to publish the study along with the patient consent details (already provided).

Are enough details provided of any physical examination and diagnostic tests, treatment given and outcomes?

Yes

Is the case presented with sufficient detail to be useful for other practitioners?

Yes

Is sufficient discussion included of the importance of the findings and their relevance to future understanding of disease processes, diagnosis or treatment?

Yes

Is the background of the case’s history and progression described in sufficient detail?

Yes

Reviewer Expertise:

Reproductive Endocrinology

We confirm that we have read this submission and believe that we have an appropriate level of expertise to confirm that it is of an acceptable scientific standard, however we have significant reservations, as outlined above.

# Response

**Published:** 2020-11-10

**Authors:** Inji Yeo

**Affiliations:** Hallym University Medical Center, South Korea

Thank you for your very informative and appropriate comments. 

**Reviewer's comment 1** : **Abstract**

 -> We added her post-operative progress, and changed the form of the last sentence not to reads repetitive

**Reviewer's comment 2: Introduction**

 -> We added more contents about objective of this article

**Reviewer's comment 3: Discussion**

 -> We added contents about normal uterine structures as commented

# Peer review: Reviewer response for version 1

**DOI:** 10.5256/f1000research.28649.r73429

**Published:** 2020-10-22

**Authors:** Jin-Sung Yuk

**Affiliations:** 1 Department of Obstetrics and Gynecology, Sanggye Paik Hospital, School of Medicine, Inje University, Seoul, South Korea

The second paragraph on page 1: it would be helpful to first explain the relationship between the bicornuate uterus and the uterine rupture in the third sentence.

 Figure 4 on page 4: The anatomical structure of the photograph cannot be distinguished. I think it will be helpful to put text on the picture to distinguish it.

 The first paragraph of the discussion: It is very similar to the first paragraph of the introduction. Variations such as changing the form of the sentence or describing the content in more detail are needed.

 The 3rd paragraph o discussion: "rupture of the superficial overlying vessels" is the result, not the cause of rupture of leiomyoma.

 The 4th paragraph on discussion: These sentences ("Uterine rupture with ~ by trauma.") fit the previous paragraph rather than this one. It would be nice to integrate with the previous paragraph and reduce the content.

 The 4th paragraph on page 5: In the conclusion of the last paragraph, it was argued that CT is helpful when sono cannot discriminate. Write something to support this.

 For example, "CT is superior than sono in circumstances that ~~~".

 The 7th paragraph on page 5: This sentence ("In our patient~~or degenerated myoma.) appears to be the result of the MRI reading. From the point of view, it would be better to move it backwards than the CT at the end of this paragraph.

 The 7th paragraph on page 5: In the last sentence ("CT imaging revealed~ PET/CT scan)", it is unclear why the MRI and PET/CT scans were performed. (bicornuate uterus? The distinction between leiomyoma and sarcoma? The distinction between rupture?)

Are enough details provided of any physical examination and diagnostic tests, treatment given and outcomes?

Yes

Is the case presented with sufficient detail to be useful for other practitioners?

Yes

Is sufficient discussion included of the importance of the findings and their relevance to future understanding of disease processes, diagnosis or treatment?

Partly

Is the background of the case’s history and progression described in sufficient detail?

Yes

Reviewer Expertise:

Endocrinologic Gynecologist

I confirm that I have read this submission and believe that I have an appropriate level of expertise to confirm that it is of an acceptable scientific standard, however I have significant reservations, as outlined above.

# Response

**Published:** 2020-11-10

**Authors:** Inji Yeo

**Affiliations:** Hallym University Medical Center, South Korea

Thank you for your very informative and appropriate comments.

**Reviewer's comment 1: The second paragraph on page 1**

 -> ****we changed the order of sentences to explain the relationship between the bicornuate uterus and the uterine rupture

**Reviewer's comment 2: Figure 4 on page 4**

 -> we put text on picture, and added description

**Reviewer's comment 3: The first paragraph of the discussion**

 -> We added normal structure of uterus, and changed the form as commented

**Reviewer's comment 4:** **The 3rd paragraph of discussion**

 -> we removed that sentence

**Reviewer's comment 5: The 4th paragraph on discussion**

 -> We changed the order of sentence, and integrated the 4 ^th^ paragraph with 5 ^th^ paragraph

**Reviewer's comment 6: The 4th paragraph on page 5**

 ->  we removed the sentence that is in argue, and added pros of CT over sono.

**Reviewer's comment 7: The 7th paragraph on page 5:**

 -> we moved above sentence backward to the end of paragraph about MRI reading

**Reviewer's comment 8: The 7th paragraph on page 5**

 -> We performed the PET/CT to make distinction between leiomyoma and sarcoma before surgery.
